# Maternal serum but not breast milk IL-5, IL-6, and IL-13 immune markers are associated with scratching among infants

**DOI:** 10.1186/s13223-016-0129-x

**Published:** 2016-05-24

**Authors:** Nelís Soto-Ramírez, Keith Boyd, Hongmei Zhang, Venugopal Gangur, Laura Goetzl, Wilfried Karmaus

**Affiliations:** Division of Epidemiology, Biostatistics, and Environmental Health, School of Public Health, University of Memphis, 236A Robison Hall, Memphis, TN 38152 USA; Department of Epidemiology and Biostatistics, University of South Carolina, Columbia, SC USA; Food Allergy and Immunology Laboratory, Michigan State University, East Lansing, MI USA; Department of Obstetrics and Gynecology, School of Medicine, Temple University, Philadelphia, USA

**Keywords:** Breast milk, Immune markers, Maternal serum, Scratching episodes, IL-5, IL-6, IL-13

## Abstract

**Background:**

Scratching in infants is considered to be related to early development of eczema. Little is known about the effects of maternal immune markers on scratching among infants. The objective is to compare the risks related to maternal serum immune markers (IMs) during pregnancy and IMs in breast milk for the occurrence of scratching in infants at 6 and 12 months of age.

**Methods:**

Pregnant women were recruited in Columbia and Charleston, South Carolina. Blood (median 3 weeks prepartum) and breast milk (3 weeks postpartum) samples were collected. The concentrations of interferon (IFN)-γ, IFN gamma-induced protein 10 (IP-10) (or CXCL10), CCL11, interleukin (IL) 1β, IL-4, IL-5, IL-6, IL-8 (CXCL8), IL-10, IL-12 (p70), IL-13, transforming growth factor (TGF)-β1, and immunoglobulin (Ig) A in both maternal serum and whey were assayed using optimized immunoassays. Scratching and skin manifestations were ascertained at 6 and 12 months. Generalized estimating equations were used to estimate relative risks (RRs) of IMs for repeated measurements of scratching, considering intra-individual correlations and adjusting for confounders.

**Results:**

Of 178 women, 161 provided blood and 115 breast milk samples. IL-1β, IL-4, IL-10, IL-12, and CCL11 in maternal serum and whey were not analyzed due to a large proportion of non-detectable values. Infants in the highest tertile of IL-6 and IL-13 in maternal serum were at higher risk of scratching (RR 1.73 and 1.84, respectively; p ≤ 0.002) compared to infants in the first tertile; similarly, infants born to mothers with high (versus low) levels of serum IL-5 were also at increased risk (RR 1.60, p = 0.002). None of the breast milk IMs studied were associated with scratching.

**Conclusions:**

Scratching but not doctors diagnosed eczema was associated with higher levels of maternal IL-5, IL-6, and IL-13 during pregnancy. Further investigations are necessary to determine how maternal serum IMs influence infants scratching.

## Background

Itching, also known as pruritus, is a symptom of a number of skin diseases including atopic dermatitis. It typically provokes scratching behavior with chronic scratching associated with high intensity of itching sensations [1–3]. Besides atopic dermatitis, dry skin, non-atopic eczema, non-atopic dermatitis, hives, ureamic kidney disorders (e.g. uremic pruritus) and fungal skin infections are typical causes that trigger itching/scratching behavior. Although it is well-known that the sensation of itch can be mediated by histamine, which is synthesized and stored in mast cells, non-histamine pathways have also been identified [1–3]. Recent elegant studies demonstrate the complex interplay of keratinocytes, immune cells, and the neurons in itch/scratching biology [4–7]. Multiple molecular mediators released by these cells are involved in the generation of itching sensation and the consequent scratching responses [3].

Itching symptoms in infancy are associated with early development of eczema [8]. According to the 2007 National Survey of Children’s Health, 12.2 % children under age 17 years have been diagnosed with eczema or any other kind of skin allergy. Interestingly among patients with eczema, 90 % experience the onset of skin disease prior to the age of 5 years [9]. The etiology of skin allergy in infancy remain unclear and the role of immune markers (IMs) of maternal origin in affecting them is controversial [10, 11]. However, little is known about the relationship between maternal serum vs breast milk IMs and infants scratching behaviour. In contrast to itching, which cannot be communicated by infants, scratching behavior is considered as a major observable symptom of eczema in infants [12].

The first environment of the fetus is the gestational environment created by its mother [13]. Outside the womb, exposure to the ‘maternal environment’ is continued through breastfeeding. The infants immune system is immature at birth; it is generally thought that the newborn rapidly develops a more mature immune system when exposed to its new environment; and during this transition, maternal immune factors derived during gestation and from breast milk are considered to play critical protective roles against infants disease susceptibility [13]. However, inconsistent results regarding the protective effect of breastfeeding on the development of eczema have been reported. Thus, whereas some studies report that breastfeeding appears to protect against eczema [14–16], others found increased risk [17–19], while some others found lack of significant association [20]. The main limitation of these studies is that they did not control for immune markers in breast milk.

Maternal blood and breast milk contain an array of immune markers, including immunoglobulins, antimicrobial factors, immune modulators including cytokines such as interferons (IFN), interleukins (IL) and chemokines [21]. These factors are thought to strengthen the infant’s immune system to protect it from infectious diseases [22]. However, it has been proposed that these IMs may also influence the susceptibility for non-infectious chronic conditions such as eczema symptoms [23]. Some IMs in breast milk (such as IgA, TGF-β1, IFN-γ) are consider to confer protection [24]. However, prenatal conditions should also be considered. Recently, we found that maternal serum and breast milk whey levels of IL-5 and IL-13 are risk markers for asthma-like symptoms among children; whereas whey IgA and TGF-β1 were protective by diminishing the relative risk of asthma-like symptoms [25].

Itch can be transmitted by activation of unmyelinated chemonociceptors, which are followed by neuropeptide release (from sensory nerves or indirectly by stimulating mediators from mast cells or keratinocytes) [26]. These neuropeptides release the histamine (from mast cells or basophils), which provokes a local inflammation, and subsequently generate the sensation of itching [2, 26]. Other mediators include proteinases, interleukins, opioids, serotonin, and vanilloid receptors [2].

Currently no previous studies have reported analyzing the relationship of immune components in maternal blood serum and breast milk whey with the development of scratching behavior among infants, which is the focus of our study. To our knowledge, this is the only study exploring the role of maternal serum in addition to breast milk whey on scratching episodes in infants. Although the sensation of itching (pruritus) cannot be reported by infants, it is in considered as a criterion for atopic dermatitis or eczema in infancy [27]. In this epidemiological study, we had to focus on observable outcomes. Hence, the primary outcome of this study was maternal observation of infant scratching. As a secondary outcome, we included physician reported eczema. We hypothesized that children whose mothers have higher levels of Th2 IMs (IL-4, IL-5, IL-13, and CCL11) in both serum and breast milk whey are more like to exhibit scratching behavior during infancy. We report that scratching behavior but not doctors diagnosed eczema among infants was associated with higher levels of maternal serum but not breast milk IL-5, IL-6, and IL-13 cytokines.

## Methods

### Study design

This study was derived from two longitudinal studies, which were approved by the University of South Carolina, the Medical University of South Carolina, and the Palmetto Health Institutional Review Boards for Human Subjects. All participants signed a written consent form either in English or Spanish.

### Participants

Expecting mothers were enrolled between April 2008 and January 2010 in Columbia and Charleston, South Carolina in obstetric clinics and prenatal classes. Eligibility criteria included (1) aged 18 years or older, (2) no chronic illness (diabetes, thyroid or adrenal disorders, or chronic infections), (3) planned to stay in the area for at least 9 months, and (4) willingness to provide a breast milk sample 2 weeks after delivery.

### Clinical data collection and outcome

Data collection was described elsewhere [25]. In brief, women took part in four telephone or in-person interviews: a core demographic and baseline interview conducted before delivery, and three interviews at 2 weeks, and 6 and 12 months after delivery, respectively. In the baseline interview, information was obtained about women’s race (African American, European American or other), maternal age, marital status, cigarette use prior to and since the beginning of pregnancy, whether cigarettes were smoked inside the home (categorized into yes vs no), education level (less than high school, some college, college graduate, or graduate school). In addition, maternal and paternal history of asthma, wheezing and whistling in the chest was obtained by asking: “Have you ever had asthma?” and “Have you ever had wheezing or whistling in the chest at any time in the past?” A history of allergic rhinitis was assessed by two questions: “Have you ever had hay fever?” and “Have you ever suffered—in the absence of a cold—from an itchy stuffy or runny nose and/or swollen, itchy eyes?” Eczema history was assessed by the question: “Have you ever had an itchy rash, which was coming and going for at least 6 months?”

The second interview was conducted 2–4 weeks after delivery to collect data on the specifics of delivery (spontaneous vaginal delivery, after induction vaginal delivery, Cesarean section), gestational age (weeks), maternal and household smoking during pregnancy period (yes, no), gender of the offspring, maternal use of antibiotics and acetaminophen during pregnancy, and history of vaginal infections/pelvic conditions during pregnancy. The date of delivery was used to define exposure to pollen in South Carolina [25].

The third and fourth interviews were based on the International Study of Asthma and Allergies in Childhood (ISAAC) questionnaire [28] and additionally ascertained scratching at ages 6 and 12 months. The recall period was restricted to the respective previous 6 months. At ages 6 and/or 12 months scratching (dichotomized) was based on a positive answer to the following question: “How often has your child scratched his/herself in the last 6 months?” [options: almost always; very often; sometimes; never]. In addition we collected information on a reported doctor’s diagnosis of eczema and eczema-like manifestations. A diagnosis of eczema was ascertained by using the question “In the last 6 months, was a doctor’s diagnosis made in your child of atopic dermatitis or eczema?” Regarding other skin manifestations of the child in the last 6 months, the following question was asked: “Have you noticed one or more of the following skin disorders in your child (1) cradle cap, (2) cheek-eczema, (3) ear eczema, and (4) eczema at other sites of the body. In addition, the interview collected information on duration of breastfeeding (weeks) as well as information on smoking in the house and pet exposure in the last 6 months.

### Blood and breast milk collection and sample preparation

Women were asked to provide one blood sample before delivery and one breast milk sample about 2 weeks after delivery. Ten milliliters of blood were taken by venipuncture from each woman in the last trimester of pregnancy (range 0–13 weeks before delivery, median 3 weeks before delivery). All serum samples were collected in sterile tubes (BD Vacutainer®, 10 mL), and centrifuged within 1 h of collection at 3500 revolutions per minute (rpm) for 10 min (minutes) at 4 °C. The separated serum samples were stored at −20 °C then after 24 h were transferred to −80 °C where the samples were stored until needed for analysis.

All women followed a detailed breast milk collection protocol. Each participant expressed approximately 15 mL of breast milk, on average 3 weeks after delivery (range 1–8 weeks), in the morning after putting the baby to breast using an electric breast pump provided by the study. All breast milk samples were collected in sterile plastic bottles (Medela 80 ml [2.7 oz]). Research staffs picked the breast milk sample up at the participant residence or a place of convenience. Within 1 h of collection, all samples were transferred to sterile centrifuges tubes and spun at 2900 rpm for 10 min at 4 °C. Fat was removed; centrifugation and fat removal steps were repeated until all fat was taken out. Finally, the cell pellet was removed. The isolated whey and serum were aliquoted and stored in a −80 °C freezer until preparation for immunoassays.

### Immuno-assay protocols

The concentrations of IL-1β, IL-4, IL-5, IL-6, IL-8 (CXCL8), IL-10, IL-12 (p70), IL-13, CXCL10 (IP-10), CCL11 (eotaxin-1), and IFN-γ in both serum and whey were assayed using the Bioplex Protein Array system (Bio-Rad Laboratories, Inc., Hercules, CA, USA). This multiplexes system allows for the assessment of several IMs in the same Bio-Rad custom-made bioplex pro human cytokine, chemokine, and growth factor multiplex plate. ELISAs (enzyme-linked immunosorbent assay) were used to determine concentrations of IgA (Immunology Consultants Laboratory, Portland, OR, USA) and TGF-β1 (R&D Systems, Minneapolis, MN, USA). To activate latent TGF-β1 to immuno-reactive TGF-β1 detectable by the Quantikine TGF-β1 immunoassay, we followed the manufacture procedure. All assays were conducted according to the manufacturer’s kit instructions. Each sample, including standards and the blank, was assayed in duplicate.

A total of 15 multiplexes and ELISAs were performed to determine the concentration of the IMs in serum and whey. For each plate we determined the limit of detection (LOD) by multiplying the standard deviation of the blank by three. Those samples that had concentrations below the detection limit were assigned a value corresponding to half the LOD.

### Exposures

The exposures were IMs levels in maternal serum before delivery and in whey. The following eight IMs were assessed: T-helper type 1/pro-inflammatory cytokines/chemokines (IFN-γ, CXCL10, IL-6, and CXCL8), T-helper type 2/pro-allergic cytokines (IL-5 and IL-13), T-regulatory/anti-inflammatory cytokine (TGF-β1) and IgA levels [25]. The concentrations were recorded in pg/mL, except for IgA, which was measured in mg/mL. Due to their distributions, we categorized IL-6, CXCL8, IL-13, IgA, and TGF-β1 into tertiles (high, median vs low) and IL-5, IFN-γ, and CXCL10 in both serum and whey, which were dichotomized (high vs median and low). Either the rank procedure was used to group IMs into tertiles, or the IMs were dichotomized (low vs high). Hence the lower group contains a combination of low values, the imputed ½ LOD values and other lower values.

### Data analysis

We ran separate analyses for maternal serum and breast milk IMs. Confounders controlled for in the statistical analyses included maternal characteristics such as race, age at pregnancy, marital status, smoking during pregnancy, household cigarette use at ages 6 and 12 months, maternal and paternal history of eczema, consumption of acetaminophen during pregnancy, and vaginal or urinary infections during pregnancy. Offspring confounders comprised of gender and season of birth. Of note, we did not control for breastfeeding duration since the IMs are intervening variables between breastfeeding and scratching (i.e., only those infants who are breastfed will receive IMs in breast milk).

Regarding repeated measurement of scratching at ages 6 and 12 months, generalized estimating equations (GEE) were employed to determine the role of the maternal IMs. Adjusting for within-participant effects using the regular maximum likelihood method, we started with an unstructured covariance matrix, which requires the least amount of constraints. Other covariance matrices, including compound symmetry and autoregressive, were considered and most of the models presented the same QIC goodness of fit statistic [29].

A total of 16 adjusted models (eight for serum and eight whey markers) were run to analyze the effect of IMs on scratching. Initially, we considered analyzing serum and whey IMs in one model; however, this led to collinearity problems [30], since most of the IMs presented correlations above 0.5 (e.g., CXCL10 and CXCL8 in whey r^2^ = 0.67; p < 0.0001).

We may consider that immune markers (IMs) in whey (after delivery) may result from maternal immune responses indicated by immune markers in maternal serum measured earlier during pregnancy. In this case, IMs in whey are the consequence of IMs in serum. Hence, they are in the same pathway with IM_whey_ as intervening variable: IM_serum_ → IM_whey_ → scratching. However, in regression models, it is not appropriate to include both serum IM and whey IM in the same model, since controlling for an intervening variable as a confounder would split the initial association between the risk factor and the outcome into two associations, destroying the potential pathway. For these reasons, we statistically analyzed IMs in serum and whey separately. Although whey IM did not gain statistical significance for scratching, they may gain importance in path-analytical models.

We detected that the IMs had non-linear relations with scratching. We tested for linearity by cross-tabulating groups with increasing immune marker levels with the outcome. Allowing non-linear associations, we therefore analyzed five IMs by comparing risk ratio (RR) estimates for scratching, contrasting the upper tertiles with the lowest tertile (low concentration) as reference group (IL-6, CXCL8, IL-13, IgA, and TGF-β1). IL-5, IFN-γ, and CXCL10 in both serum and whey were dichotomized (high vs median and low). Also we assessed the use of quartiles; however, there were not enough observations per category due to the sample size. Since we ran 16 individual adjusted models, we applied false discovery rate (FDR) (p = 0.05) for serum and whey IMs to adjust for multiple testing and avoid false positive associations [31]. We estimated the 95 % confidence interval (CI) and also presented p-values to identify associations that survive penalizing for multiple testing.

All confounders were simultaneously entered as indicator variables into the models. A backward elimination process was used to retain confounders in the final model: confounders were those variables which changed the effect of the main association by 10 % or more when adding this factor into the model. All statistical analyses were performed using SAS version 9.3.

## Results

Of 178 women who participated, 161 provided maternal blood and 115 breast milk samples (98 provided both prenatal blood sample and breast milk sample). Most of the participants were well educated and European American (Table 1). Approximately 84 % of the mothers and fathers were living together (i.e., married and single, but living together). A history of maternal eczema was reported by 8.8 %, a history of asthma by 27.7 %, and a history of rhinitis by 49.6 %. Of the mothers 5.9 % reported smoking during pregnancy, and 44.1 % had vaginal or urinary infections during pregnancy. The average mother’s age was 30 years.

**Table 1:** Characteristics of the participants who have information on child’s scratch status either at 6 or 12 months (n = 140)

Variables	n (%)
Maternal race	
African American (AA)	30 (21.7)
European American or Other	108 (78.3)
Sex of the infant	
Male	70 (50.0)
Maternal smoking during pregnancy	8 (5.9)
Smoke 6 months after delivery	14 (10.6)
Smoke 12 months after delivery	15 (15.0)
History of parental allergy	
Asthma	
Mother	38 (27.7)
Father	35 (21.21)
Eczema	
Mother	12 (8.8)
Father	6 (4.1)
Rhinitis	
Mother	68 (49.6)
Father	77 (46.7)
Marital status	
Married	97 (71.9)
Other	1 (0.7)
Single but living together	16 (11.9)
Single	21 (15.6)
Pets 6 months after delivery	4 (3.0)
Pets 12 months after delivery	6 (5.9)
Season of child’s birth	
Fall	30 (22.1)
Spring	30 (22.1)
Summer	30 (22.1)
Winter	46 (33.7)
Use of antibiotics during pregnancy	34 (19.1)
Vaginal/urinary infections during pregnancy^a^	60 (44.1)
Method of delivery	
Spontaneous vaginal delivery	32 (41.0)
After induction vaginal delivery	26 (33.3)
Cesarean section	20 (25.6)
Fish intake during pregnancy	6 (7.6)
Education	
Less than high school	11 (8.0)
Some college	33 (23.9)
College graduate	45 (32.6)
Graduate school	49 (35.5)
Tylenol intake during pregnancy	58 (41.4)
	Mean (n; 5, 95 %)
Maternal age during pregnancy	29.9 (131; 19.7, 39.0)
Gestational age (weeks)	38.6 (135; 35.0, 41.0)

^a^Including urinary tract infection, vaginitis or vaginosis, genital warts, genital herpes, gonorrhea, syphilis, chlamydia, trichomoniasis, yeast infection, and group B *Streptococcus* infections

At 6 months of age 72.8 % of the mothers reported that they observed their child scratching in the last 6 months and 66.7 % at age 12 months covering the period from 7–12 months (Table 2). The prevalence of doctor’s diagnosis of eczema was lower: 22.6 % at age 6 months and 25.5 % at 12 months.

**Table 2:** Scratching episodes and other skin manifestation observed by the mother in the first 6 months and from 7–12 months

	First 6 months, n = 136 (%)	7–12 months, n = 102 (%)
Scratching episodes	72.8	66.7
Doctor’s diagnosis of eczema	22.6	25.5
Eczema-like symptoms	33.8	35.3
Cheek-eczema	22.1	18.6
Eczema on other sites of the head and body	24.3	16.7
Ear eczema	5.9	9.8
Swollen lips	1.5	0
Sore nappy-area	2.9	2.9
Cradle cap	21.3	16.7

In the first 6 months, any eczema at any location was reported by 33.8 % of the mothers, and in 35.3 % from month 7 to 12. Among specific eczema locations, cheek eczema was most prevalent. Also cradle cap was a prevalent skin symptom reported in children (29.9 %; p < 0.05). Regarding scratching, doctors diagnosis of eczema was not related to scratching in the first 6 months and weakly between 7 and 12 months (Table 3). Cradle cap showed the strongest association (25.0 %; p < 0.05). Between 7–12 months, reported scratching was associated with ear and cheek eczema.

**Table 3:** The associations of reported scratching with reported eczema diagnosis at ages 6 months

	Reported scratching in the first 6 months of age		Reported scratching from 7–12 months of age	
	Yes, n = 99 (%)	No, n = 37 (%)	Yes, n = 68 (%)	No, n = 34 (%)
Doctor’s diagnosis of eczema	22.2	24.3	30.9^#^	14.7
Eczema-like symptoms	35.4	29.7	41.2	23.5
Cheek eczema	23.3	18.9	23.5^#^	8.8
Ear eczema	6.1	5.4	14.7*	0
Eczema on other sites	24.2	24.3	16.2	17.7
Cradle cap	29.9*	0	25.0*	0

* p < 0.05

^#^ p < 0.1

Next, we assessed the associations between IMs in maternal serum and whey with repeated observations of scratching at ages 6 and 12 months controlling for confounders (Table 4). Infants exposed to higher levels of Th-2 cytokines (IL-5 and IL-13) and pro-inflammatory cytokine IL-6 in serum had a significantly higher relative risk for scratching (RR 1.73, 1.60, 1.84; respectively) (Table 4). A linear trend was observed for IL-6 and IL-13 in serum on scratching in infants. After adjusting for multiple testing, no whey IM was statistically significant.

**Table 4:** Adjusted effects of IMs in maternal serum and in breast milk whey on mothers’ observation of scratching (1 = yes or 0 = no) at ages 6 and 12 months (n = 156 and 169 observations, respectively)

IMs in maternal serum before delivery					IMs in breast milk whey			
Immune Marker^b^	Levels	RR^a^ 95 % CI	p value^c^	FDR^c^	Levels	RR^a^ 95 % CI	p value^c^	FDR^c^
T-helper type 1/pro-inflammatory cytokines/chemokines (pg/mL)								
INF-γ	≥5.92	1.10 (0.88, 1.36)	0.37	0.52	≥2.13	1.24 (0.94, 1.61)	0.11	0.41
	Reference <5.92				Reference <2.13			
CXCL10	≥202	1.28^c^ (1.03, 1.58)	0.02	0.06	≥338	0.88 (0.67, 1.17)	0.40	0.64
	Reference <202				Reference <338			
IL-6	≥4.25	1.73^c^ (1.28, 2.31)	0.0003	0.002	≥2.12	0.91 (0.94, 1.43)	0.67	0.9
	2.12–4.25	1.44 (1.05, 1.95)	0.02	0.06	2.12	1.28 (0.73, 2.24)	0.37	0.64
	Reference <2.12				Reference <2.12			
CXCL8	≥1.59	1.37^c^ (0.99, 1.91)	0.05	0.06	≥5.28	0.77 (0.48, 1.23)	0.28	0.13
	0.81–1.59	1.22 (0.94, 1.61)	0.13	0.15	1.40–5.28	0.81 (0.52, 1.25)	0.36	0.93
	Reference <0.81				Reference <1.40			
T-helper type 2/pro-allergic cytokines/chemokines (pg/mL)								
IL-5	≥1.59	1.60^c^ (1.24, 2.07)	0.0002	0.002	≥0.46	1.22 (0.88, 1.69)	0.22	0.60
	Reference <1.59				Reference <0.46			
IL-13	≥1.85	1.84^c^ (1.46, 2.29)	<0.0001	0.0001	≥1.71	1.47 (1.06, 2.02)	0.01	0.13
	0.13–1.85	1.30 (0.96, 1.75)	0.08	0.15	0.11–1.71	0.82 (0.57, 1.19)	0.31	0.63
	Reference <0.13				Reference <0.11			
T-regulatory/anti-inflammatory cytokines (pg/mL)								
TGF-β1	≥28,809.27	1.12 (0.70, 1.80)	0.61	0.73	≥774.63	0.71 (0.48, 1.05)	0.09	0.41
	16,430.59–28,809.27	0.82 (0.52, 1.29)	0.40	0.52	438.28–774.63	0.98 (0.73, 1.31)	0.91	0.93
	Reference <16,430.59				Reference <436.28			
Secretory immunoglobulin A (mg/mL)								
IgA	≥6.02	0.96 (0.65, 1.40)	0.83	0.88	≥87.58	0.98 (0.66, 1.44)	0.93	0.93
	4.06–6.02	0.97 (0.63, 1.46)	0.87	0.88	1.28–87.58	1.07 (0.76, 1.47)	0.71	0.90
	Reference <4.07				Reference <1.28			

^a^IMs were categorized into tertiles levels using the lowest level as reference, except for IL-5, IFN-γ, and CXCL10 in both serum and whey, which were dichotomized (high vs median and low). All IMs in serum and whey were adjusted for child’s sex, maternal race and age, vaginal or urinary infections during pregnancy, parental history of eczema, consumption of acetaminophen during pregnancy, marital status, season of child’s birth, smoking during pregnancy, and household cigarette use at ages 6 and 12 months

^b^Maternal education, parental history of rhinitis and asthma, pets, gestational age, maternal antibiotics consumption, and mode of delivery were removed from the models because they were not confounding

^c^The overall effect of the level was statistically significant after applying a false discovery rate (adjusted p value <0.05)

Regarding the covariates, maternal education, gestational age, parental history of rhinitis and asthma, pets, maternal antibiotics consumption, and mode of delivery were removed from the models because they were not confounding. Infants whose mothers were African American, were at higher risk for developing scratching episodes than children of a European American mother (RR 1.31; 95 % CI 1.02, 1.68; p = 0.03). In addition, infants of single mothers were more at risk for developing scratching than infants of married mothers (RR 1.46; 95 % CI 1.04, 2.06; p = 0.02). Regarding infant sex, boys presented more scratching episodes than girls (RR 1.29; 95 % CI 1.02, 1.63; p = 0.02).

## Discussion

This is the first study that simultaneously examines the effect of immune markers (IMs) in both maternal serum and breast milk whey on scratching episodes in infants at ages 6 and 12 months. We found that three IMs out of serveral markers studied in maternal serum were associated with scratching in infants. Our results reveals an increase in the risk of scratching when infants (as fetuses) are exposed to high levels of IL-6, IL-5, and IL-13 cytokines in maternal serum, but not in breast milk whey. Regarding confounders: maternal race, maternal marital status, and child’s sex were all linked to scratching.

The strengths of this study include the prospective design, the independent collection and analysis of clinical data and maternal serum and breast milk IMs. Our study is one of few comparable studies with a sample size of 100 and more [13]. With regards to selection bias, participation in studies during pregnancy and infancy depends on volunteering and a high level of dedication to study requirements. We enrolled 231 women and received breast milk or blood samples from 178 participants (77.1 %). In addition, we used statistical techniques to control for potential confounders.

Also, since levels of IMs in whey may vary over time, we tested whether the immune mediators’ levels correlated with the interval of collection after birth. None of the IMs were correlated with the time of milk collection, which is an agreement with a recent review on breast milk IMs [13]. Regarding the dates of maternal blood sample collection, most IMs except for IFN-γ were not correlated with the number of days of blood collection before delivery. Since IFN-γ was not associated with scratching, there was no need to control for the dates of maternal blood sample collection in the other explanatory models.

Some IMs were not included in the analysis due to a large proportion of non-detectable values. Different factors may have contributed to the lack of detection. These may include the handling and temperature the extracted breast milk was stored prior to the assay. Since breast milk may have been left at room temperature for about an hour after expression, proteases in breast milk may have degraded the IMs in whey. In addition, cytokines in breast milk may have been undetectable due to their short half lives.

A limitation of our study is that models for each IM were run independently. This approach has the drawbacks missing possible interactions between IMs; however, models that included possible interactions would have required a much larger sample size to detect significant effects. Also, if mutual adjustments were made for immune markers, correlations between the immune markers would have resulted in collinearity between various IMs and thus would have produced biased risk estimates. However, to adjust for multiple testing of various single IMs, the false discovery rate was used.

In addition to main effects, also an imbalance of Th1 and Th2 immune markers in maternal serum and in breast milk whey may be considered as risk factors. However, regarding the sample size, our study was not designed to also assess ratios of immune markers as predictors of scratching episodes, only to investigate the main role of IMs in maternal serum and breast milk whey. Therefore we did not analyze whether an imbalance of multiple pro-inflammatory and anti-inflammatory IMs are related to scratching episodes. Analyses with inclusion of all ratios in the regression models cannot be conducted with sufficient statistical power. Hence we recommend future studies with a larger sample size to address these associations.

### T-helper 2 or type 2 cytokines

Our results suggest that high levels of two Th-2 or type 2 immune response markers (IL-5 and IL-13) in maternal systemic circulation (serum) were a risk for the occurrence of scratching in infants 6–12 months later. Th2 cells play a critical role in the pathogenesis of allergic diseases including atopic dermatitis [32]. The Th2 cells are critical players in allergy because: (1) IgE antibody responses are Th2 (i.e., IL-4) dependent; (2) IL-5 is critical player in eosinophilia; (3) IL-13 is critical in mucus production and also enhances IgE responses; (4) these cytokines can also be produced by mast cells, basophil and eosinophil that can result in positive feed-back enhancement of chronic allergic reactions such as atopic dermatitis [33]. Our novel findings that higher maternal circulating levels of IL-5 and IL-13 cytokines enhances risk of scratching in infants reflects the possibility that such maternal environment conditions the fetus for a future disease phenotype upon delivery [23, 33, 34]. Prior investigations suggest that maternal cytokines play a role in the fetal programming, with a significant impact on fetal immune outcomes [34].

### T-helper-1 or type 1 and pro-inflammatory cytokines

IMs related to T helper 1 (Th1) or type 1 immune responses are linked to cell-mediated and phagocyte-dependent protective responses and are generally considered as anti-allergic immune responses; it was surprising that we did not find a significant association of prototypic Th1 cytokine IFN-λ in the maternal circulation with protection from scratching among infants; however a type 1 promoting chemokine, CXCL10 (or IP-10) showed weak association before FDR analysis (Table 4) [35, 36].

Recent studies have identified IL-31 and IL-33 as a critical pruritogenic cytokines in humans and in mice because over expression of IL-31 and IL-33 in the skin enhances pruritus and atopic dermatitis phenotypes in mice [5]; and atopic skin lesions in both humans and in mice express very high levels of these two cytokines [4]. Most importantly, studies show that IL-33 positively interacts with IL-5 and IL-13 in exhibiting its pruritogenic function [5]. Here we report a significant relationship of maternal Th2 cytokines IL-5/IL-13 with infant scratching behavior. This suggest that future work is needed to evaluate whether maternal levels of pruritogenic cytokines IL-33 (and IL-31) could also be critical risk factors for infant scratching [4–6, 37].

We unexpectedly found that the prototypic pro-inflammatory cytokine IL-6 in the maternal circulation was highly associated with scratching behavior among infants. Results of the repeated measurements suggest a risk for scratching 6–12 month later when the levels of IL-6 in maternal serum were higher. We showed that IL-6 cytokine in serum correlate positively with type 2 markers (IL-13, IL-5) and negatively with TGF-β1 [25]. To the best of our knowledge, no studies have considered the effect of this combination of IMs in maternal serum on scratching episodes. Furthermore, IL-6 has not been reported to be a critical player in allergic disease in general although a recent report has linked elevated cardiac IL-6 levels to tree nut induced systemic anaphylaxis for the first time in a mouse model [38]. Neverthess, IL-6 is considered to modulate pruritus because of its pro-inflammatory activity. For example, IL-6 levels were significantly higher in hemodialysis patients with uremic pruritus, compared to hemodialysis patients without uremic pruritus [2]. Further assessment is necessary to clarify the interrelation of IL-6 with proto-typic Th2 cytokines such as IL-5 and IL-13 on scratching and the possibility that scratching is more than simply a Th2 driven response.

### Lack of association of infant scratching with breast milk immune markers

In contrast to maternal serum Th2 cytokines and IL-6 that are linked to infant scratching behaviour in this study, no link with breastmilk IMs was found. This finding was surprising, and suggests that the half life of breast milk IM’s makes their quantification in a study such as ours difficult, or that systemic immune markers do not always correlate with mucosal (breast) immune markers.

### Associations between scratching and physician diagnosed eczema

There is a lack of agreement between reported scratching and reported doctor’s diagnosis of eczema but a strong association with cradle cap and weak associations with maternal observance of specific eczema location between 7–12 months. It is possible that the observed scratching is a response to different etiologies. Other triggering factors modulating pruritus may include contact and aero allergens, microbial agents, food, exogenous irritants, and endogenous factors [26]. However, maternal reported scratching can be considered as a more sensitive (but less specific) marker for childhood atopic dermatitis than physician diagnosed eczema. Further investigations are necessary to determine the agreement doctor’s diagnosed eczema and scratching episodes.

## Conclusion

According to our findings, Th2 related IMs in maternal serum may predispose the child to develop scratching in infancy. Hence, this may suggest that the gestational environment (IMs in maternal serum) to which the offspring is exposed in uterus, could contribute to the risk of developing immune-related conditions after birth; however further investigation is warranted. We identified that Th2 maternal serum IMs are associated to scratching episodes in children of ages 6 and 12 months. Thus, our data predicts that high maternal serum levels of IL-6, IL-5, and IL-13 during gestation are IMs that pose a higher risk for scratching among infants. Future studies should include maternal serum immune markers as predictors of scratching. In addition, further investigations are necessary to determine how maternal cytokines influence scratching and how they are related to the recently described pruritogenic cytokines such as IL-31 and IL-33.

## Abbreviations

IMs: immune markers
IL: interleukin
Ig: immunoglobulin
IFN-γ: interferon gamma
IP-10: IFN gamma-induced protein 10
TGF: transforming growth factor
Th: T helper
RR: relative risk
CI: confidence interval
